# Does Medial Overhang of the Greater Trochanter Influence Femoral Stem Position During Cemented Hip Arthroplasty? A Retrospective Radiological Review

**DOI:** 10.7759/cureus.10968

**Published:** 2020-10-15

**Authors:** Morgan Bayley, Peter Cnudde, Phillip J Adds, Stephen Jones, Rhodri L Williams

**Affiliations:** 1 Orthopaedics, Cardiff & Vale University Health Board, Cardiff, GBR; 2 Orthopaedics and Trauma, Hywel Dda University Health Board, Carmarthen, GBR; 3 Anatomy, St George's University, London, GBR; 4 Orthopaedics and Trauma, Cardiff & Vale University Health Board, Cardiff, GBR; 5 Orthopaedics and Traumatology, Hywel Dda University Health Board, Carmarthen, GBR

**Keywords:** greater trochanter, anatomy, total hip arthroplasty, x-ray

## Abstract

Purpose

We investigate the effect that variation in the anatomy of the greater trochanter (GT), in particular the medial overhang, can have on femoral stem alignment in total hip arthroplasty (THA).

Methods

Pre- and post-operative anteroposterior pelvic radiographs of 576 consecutive patients undergoing THA were retrospectively analysed. Medial overhang of the GT relative to the lateral femur diaphysis was measured. The femoral morphology was classified according to Dorr classification. The alignment of the femoral stem axes on post-operative radiographs was recorded.

Results

Following exclusions, 500 THAs performed by six surgeons all using the same cemented polish tapered stems were analysed: 320 THAs were performed via the posterior-lateral approach and 180 via the direct-lateral approach. Mean stem varus was 0.53° (range: -7 to 7°). Mean medial overhang was 21 mm (range: 8-43 mm). An overhang of <20 mm had a mean varus of -0.1°, an overhang of 20-30 mm had a mean varus of 0.8° and an overhang of >30 mm had a mean varus of 2.33°. Those with an overhang of <20 mm had a 2% chance of significant varus (≥4°), increasing to 9.5% for 20-30 mm and 44.4% for >30 mm. One-way analysis of variance comparison of these groups returned a p-value of <0.0001. Dorr type A femora had a mean varus of 0.52°, Dorr B had a mean varus of 0.54° and Dorr C had a mean varus of 0.46°. The posterior-lateral approach had a mean varus of 1.05° (range: -7 to 7°) compared to -0.40° (range: -5 to 5°) for direct-lateral approach. The t-test comparing approach was p < 0.0001.

Discussion

The extent of medial overhang of the GT can adversely affect the final stem position in THA, resulting in a statistically significant increase in mean stem varus. There is a linear relationship between stem position and GT overhang, with an increased chance of significant varus malposition (44.4% with >30 mm of overhang).

Conclusions

Scrutiny of pre-operative radiographs to determine high-risk patients is important, and we propose a classification system of GT anatomy to aid assessment.

## Introduction

Total hip arthroplasty (THA) is among the most cost-efficient procedures available in the National Health Service [1], and consequently large numbers of THAs are performed annually. In England, Wales and Northern Ireland, 97,792 patients received a primary THA in 2018 [2]. The National Joint Registry demonstrates clear trends in implant selection. All cemented prostheses accounted for 56.1% of implants in the first report, but this has fallen dramatically to 28.2%. Uncemented implants usage rose from 19.9% in 2003 to a peak of 45.8% in 2010, and has now declined to 37.8% in 2017, whereas hybrid prosthesis usage has steadily increased from 13.2% to 30.3%. Overall, 54.4% of all stems in the registry are cemented, and in the most recent report 58.5% of stems implanted were cemented [2].

Understanding proximal femoral anatomy is an important consideration in THA. There can be significant variation in anatomy of the proximal femur [3], in particular the overhanging trochanter [4]. Intra-operatively, the surgeon must ensure the prosthesis entry point to achieve satisfactory coronal alignment. We believe that a failure to recognize anatomical variations in medial overhang can result in the prosthesis being medially translated or placed in varus. A femoral stem placed in varus alters femoral offset, leg length and centre of rotation, and consequently affects hip biomechanics. Cemented femoral stems placed in greater than 5° of varus have been shown to have accelerated loosening [5] and increased revision rates [6]. It has been suggested that varus malpositioned stems create unfavourable proximal stresses on the cement mantle resulting in premature failure [7] as a result of medial midstem pivoting [8].

The seminal paper by Dorr et al. [9] described the importance of the relationship of proximal femur shape and cortical thickness for implant selection. However, to date, only one article by Grechenig et al. [4] looked specifically at the overhang of the greater trochanter. The authors photographed 100 proximal cadaveric femurs and divided them into four groups depending on their degree of overhang. Their study focused on anatomical variations relating to piriform fossa entry intramedullary nails. We feel that trochanteric overhang is also of significant clinical relevance for arthroplasty surgery, and to our knowledge there are no studies in the literature relating to this. Radiographs remain the gold standard for demonstrating structural changes of the hip because image acquisition is non-invasive, cheap, fast and generally available [10]. We therefore performed this radiological study as it is relevant to daily arthroplasty practice.

## Materials and methods

We retrospectively reviewed 576 consecutive patients who underwent THA in our unit over two years starting in January 2014. Pre-operative and post-operative anteroposterior (AP) pelvic radiographs were analysed using Synapse® radiology software (Fujifilm, Tokyo, Japan). Two investigators (M.B. and R.W.) were blinded to operating surgeon and surgical approach. Seventy-six patients were excluded, as shown in Table 1. The remaining 500 patients all received a primary THA using a cemented Exeter® tapered prosthesis (Stryker, Kalamazoo, MI, USA).

**Table 1:** Reasons for patient exclusion

Exclusions	Number
Total	576
Uncemented stem	45
Revision surgery	16
Abnormal anatomy	7
Previous fixation	4
Inadequate images	4
Remaining	500

**Figure 1:**
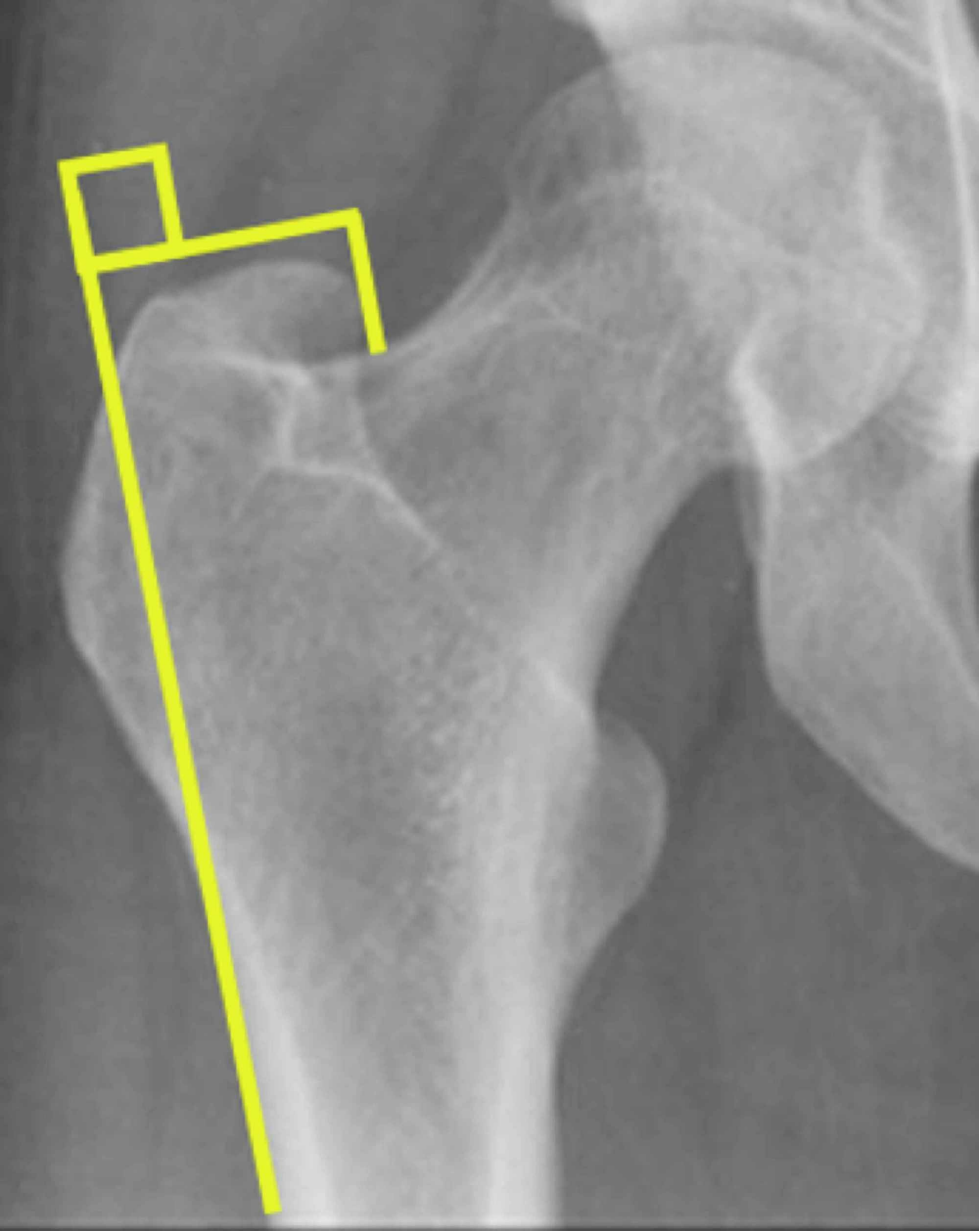
Measurement of medial overhang relative to the lateral femur diaphysis.

On the pre-operative radiograph, the medial overhang of the greater trochanter was measured relative to and 90° perpendicular to the lateral femur diaphysis (Figure 1).

Additionally, the radiographic femoral morphology was classified according to Dorr classification [9]. On the post-operative radiograph, the coronal alignment of the femoral stem axes was subsequently recorded. A perfectly neutral stem had an angle of 0°; a positive angle indicates a varus-aligned stem and a negative angle indicates a valgus-aligned stem. 

Following radiographic analysis, the unique unit number allowed the operating surgeon and surgical approach to be identified for each patient. Statistical analysis was performed using the Student t-test and analysis of variance (ANOVA) test.

## Results

Following exclusions, 500 cemented THAs performed by six consultant surgeons were analysed. The mean patient age was 69 years (range: 28-94 years), with 293 being female and 207 being male. Of these, 320 were performed through the posterior-lateral approach and 180 through the direct-lateral approach. 

The average medial overhang on the pre-operative radiographs was 21 mm (range: 8-43 mm), with a standard deviation of 5.64. The mean overhang for women was 20.2 mm compared to 22.9 mm for men. Overall, 182 patients (36.4%) had Dorr type A femora, 264 (52.8%) had Dorr type B femora and 54 (10.8%) had Dorr type C femora. 

Analysing the post-operative radiographs revealed an average stem varus of 0.53°, ranging from 7° of valgus to 7° varus with a standard deviation of 2.62.

Comparing the degree of varus for males and females did not reveal a significant difference, with men having a mean of 0.54° compared to 0.50° for women (Table 2). Comparison of Dorr classification also did not reveal a significant difference, with type A femora having a mean varus of 0.52°, type B having a mean varus of 0.54° and type C having a mean varus of 0.46°. However, comparing surgical approach, the posterior-lateral approach had a mean varus of 1.05° compared to -0.4° for the direct lateral. The Student t-test comparing the two approaches returned a significant p-value of <0.0001.

**Table 2:** Mean degree of stem varus in different groups.

Sex	Number	Mean varus
Male	207	0.54°
Female	293	0.50°
Dorr Classification		
A	182	0.52°
B	264	0.54°
C	54	0.46°
Approach		
Posterior-lateral	320	1.05°
Direct lateral	180	-0.4°

We classified the medial overhang into three groups: type 1 (small) measuring less than 20 mm, type 2 (medium) measuring 20-30 mm and type 3 (large) measuring greater than 30 mm. As the size of medial overhang increased, there was an increase in the mean stem varus. A type 1 overhang had a mean varus of -0.1°, type 2 had a mean varus of 0.8° and type 3 had a mean varus of 2.3°. ANOVA testing of the mean stem varus within these three groups returned a p-value of <0.0001.

When looking at the proportion of patients with a stem in significant varus (defined as greater than 4°), there was once again an increase within the groups. Type 1 had 2.0% of stems in significant varus compared to 9.5% of type 2 overhangs and 44.5% of the type 3 overhang group (Table 3).

**Table 3:** Classification of medial overhang and its effect on stem varus

Type	Medial overhang	Number	Mean varus	Varus > 4°
1	Small (<20 mm)	202	-0.1°	2.0%
2	Medium (20-30 mm)	262	0.8°	9.5%
3	Large (>30 mm)	36	2.3°	44.5%

## Discussion

Proximal femoral anatomy can be extremely varied, with previous authors describing significant variation in medial trochanteric overhang [3,4]. We believe this variation can influence femoral prosthesis alignment, which impacts on implant function and survivorship. We postulate that an unrecognized large medial overhang can push a cemented stem medially and into the varus during implantation. Varus malposition has been proven to result in early failure and higher revision rates [5,6].

This large study demonstrates significant variation in medial overhang, with a mean value of 21 mm, ranging from 8 to 43m m. More than half of our cohort had Dorr type B femora and one-third had Dorr type A femora.

The mean stem varus on post-operative radiographs was 0.53°, ranging from 7° of valgus to 7° of varus. Neither gender nor Dorr classification appeared to affect the degree of stem varus. We had prediction that Dorr type C “stove-pipe” femurs would have more varus stems as the capacious canals would allow more variation in implant placement; however, this was not the case.

Interestingly, the posterior-lateral approach had a statistically significant increased chance of a stem placed in varus compared to the direct lateral approach. We do not know whether this a consequence of the surgical approach or technique variation between surgeons. We do not believe that there is significant difference in visualization of the proximal femur or soft tissue interference between the two approaches.

We devised a simple classification of medial overhang: type 1 as <20 mm, type 2 as 20-30 mm and type 3 as >30 mm. Grechenig et al. [4] classified femurs into four groups, and their work seems to correlate with ours. Their group 1 indicates a minimal overhang, group 2 indicates moderate overhang and group 4 indicates the largest overhang. Furthermore, the proportion in each group is similar, with roughly 40% in the first group and the fewest in the last group.

When looking at coronal stem alignment, there is a strong statistical difference between our groups, with type 3 femurs having a greater mean stem varus. In addition, we have shown the increased chance of significant varus malposition in type 3 femurs, with 44.5% of stems being positioned in >4° of varus.

The main limitation of our study is the fact we only analysed AP radiographs, which meant not assessing the three-dimensional anatomy. In addition, an AP pelvis radiograph does not factor in femoral rotation reducing measurement precision. CT analysis would resolve these issues; however, it would expose patients to greater radiation. The use of AP radiograph is the standard modality of imaging, which makes this study highly clinically relevant. Additionally, the radiological measurements were performed by two investigators, and inter- or intra-observer variation was not formally assessed.

## Conclusions

Previous studies have assessed proximal femoral anatomy and trochanteric overhang. However, to our knowledge, this is the first study to look at medial overhang of the greater trochanter and its effect on femoral component alignment during arthroplasty surgery. We have demonstrated a significant risk of varus malposition of cemented femoral implants with increasing medial overhang. We advise scrutinizing pre-operative radiographs and using our simple classification to identify patients at risk of varus stem placement. Surgical technique should be modified to ensure adequate resection of the medial overhang facilitating lateralization and appropriate stem position in high-risk patients.

## References

[1] Jenkins PJ, Clement ND, Hamilton DF, Gaston P, Patton JT, Howie CR (2013). Predicting the cost-effectiveness of total hip and knee replacement. Bone Joint J.

[2] (2020). National Joint Registry 16th Annual Report. https://reports.njrcentre.org.uk/portals/0/pdfdownloads/njr%2016th%20annual%20report%202019.pdf.

[3] Rubin PJ, Leyvraz PF, Aubaniac JM, Argenson JN, Estève P, de Roguin B (1992). The morphology of the proximal femur. A three-dimensional radiographic analysis. Bone Joint J.

[4] Grechenig W, Pichler W, Clement H, Tesch NP, Grechenig S (2006). Anatomy of the greater femoral trochanter: clinical importance for intramedullary femoral nailing: anatomic study of 100 cadaver specimens. Acta Orthop.

[5] Ebramzadeh E, Sarmiento A, McKellop HA, Llinas A, Gogan W (1994). The cement mantle in total hip arthroplasty. Analysis of long-term radiographic results. J Bone Jt Surg.

[6] Devitt A, O’Sullivan T, Quinlan W (2016). 16- to 25-year follow-up study of cemented arthroplasty of the hip in patients aged 50 years or younger. J Arthroplasty.

[7] de Beer J, McKenzie S, Hubmann M, Petruccelli D, Winemaker M (2006). Influence of cementless femoral stems inserted in varus on functional outcome in primary total hip arthroplasty. Can J Surg.

[8] Gruen TA, McNeice GM, Amstutz HC (1979). “Modes of failure” of cemented stem-type femoral components: a radiographic analysis of loosening. Clin Orthop Relat Res.

[9] Dorr LD, Faugere MC, Mackel AM, Gruen TA, Bognar B, Malluche HH (2016). Structural and cellular assessment of bone quality of proximal femur. Bone.

[10] Kinds MB, Welsing PM, Vignon EP, Bijlsma JW, Viergever MA, Marijnissen AC, Lafeber FP (2011). A systematic review of the association between radiographic and clinical osteoarthritis of hip and knee. Osteoarthr Cartil.

